# A Rare Case of Myocarditis After the First Dose of Moderna Vaccine in a Patient With Two Previous COVID-19 Infections

**DOI:** 10.7759/cureus.24802

**Published:** 2022-05-07

**Authors:** Aniekeme S Etuk, Inimfon N Jackson, Hercules Panayiotou

**Affiliations:** 1 Internal Medicine, Thomas Hospital Infirmary Health, Fairhope, USA; 2 Internal Medicine, Einstein Medical Center Philadelphia, Philadelphia, USA; 3 Interventional Cardiology, Mobile Infirmary Medical Center, Mobile, USA

**Keywords:** post vaccination myocarditis, moderna vaccine, covid-19 infection, antigen, antibodies

## Abstract

Myocarditis is the inflammation of the cardiac muscle caused by a variety of factors ranging from infections to autoimmune diseases. Most cases of vaccine-induced myocarditis occur after the second dose of vaccination; however, a few cases have been reported following the first dose of vaccination with or without previous coronavirus disease 2019 (COVID-19) infection. A case of myocarditis occurring about three weeks after the first dose of the Moderna vaccine has been reported in a patient with one previous COVID-19 infection. However, there have not been any documented cases of myocarditis after the first dose of the Moderna vaccine in a patient with two prior COVID-19 infections. Our index patient had already experienced two COVID-19 infections in the past and was diagnosed with myocarditis eight hours after receiving the first dose of the Moderna vaccine. The susceptibility to developing this likely stems from the possible production of antibodies to the viral antigen from previous COVID-19 infections. Furthermore, the fact that our patient developed symptoms eight hours after receiving the vaccine suggests a possible additive effect of antibodies produced from the two previous COVID-19 infections. This case report suggests that individuals repeatedly infected with COVID-19 may be at increased risk of myocarditis following the administration of the Moderna vaccine.

## Introduction

Myocarditis is defined as the inflammation of the myocardium, occurring as a result of some pathologic immune changes in the heart [1-3]. These changes include alterations in the number and subtypes of lymphocytes, macrophages, and antibodies [3]. The long-term effect of these processes compromises the structure and function of the cardiomyocytes, thereby leading to impairment in either the contractile or conducting system of the heart [3]. The prevalence of myocarditis has been estimated to range from 10.2 to 105.6 per 100,000 people worldwide, and it is predominantly found among young adults of the male sex [2,4]. Myocarditis can be caused by both infectious and non-infectious agents [1,3,5], presenting with either focal or diffuse cardiac muscle involvement [3]. It has also been reported to occur as a rare complication of various coronavirus disease 2019 (COVID-19) vaccines [1,2,5,6], and among those who received the Moderna vaccine, it has been observed mostly after the second dose [6]. Also, to date, there have been a few reports of myocarditis after the first dose of the Moderna vaccine, with or without previous COVID-19 infections.

## Case presentation

An 18-year-old male with two previous episodes of COVID-19 infections, eight months and two months prior, presented with a complaint of sudden onset of chest pain that had started about eight hours after receiving the first dose of the Moderna vaccine. The patient reported it to be located in the center of his chest and rated it as a 7/10 in its worse form. He described it as being sharp, pressure-like, and squeezing, radiating to the back and worse with inhalation and coughing. The chest pain was associated with fever, cough, shortness of breath, fatigue, diarrhea, and vomiting. The patient reported a history of nose bleeding that had started about the same time and resolved without any intervention. Also, his past medical and surgical history was non-contributory, and he was not aware of any family history of cardiac diseases. On examination, the patient appeared to be an obese male in acute painful distress with a temperature of 98.2 °F, blood pressure of 128/88 mmHg, pulse rate of 88 beats per minute, respiratory rate of 18 cycles per minute, and oxygen saturation of 98% on room air. The precordium was quiet, and first and second heart sounds were heard with no added sounds. A list of differential diagnoses considered in the workup of this patient included ischemic heart disease, pleurisy, pericarditis, pulmonary embolism, pneumonia, and COVID-19.

**Figure 1:**
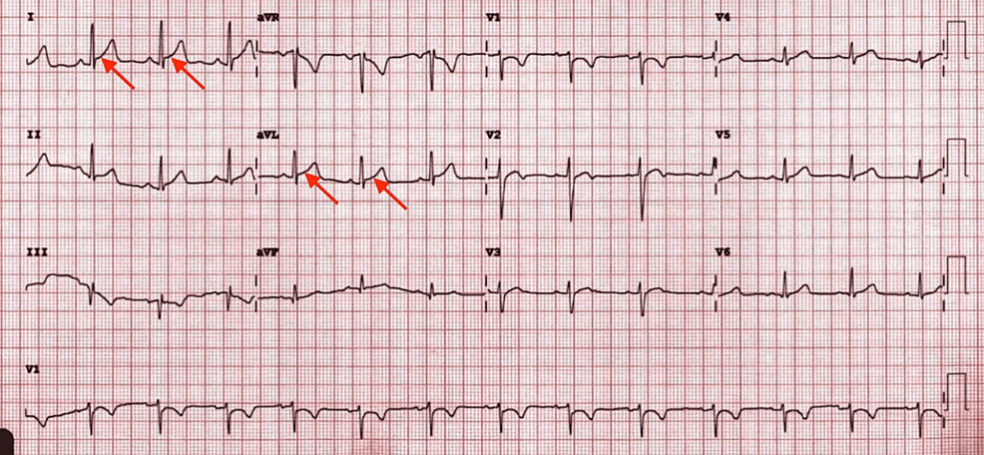
EKG showing normal sinus rhythm with ST-segment elevation in some lateral leads (I and aVL) EKG: electrocardiogram

**Figure 2:**
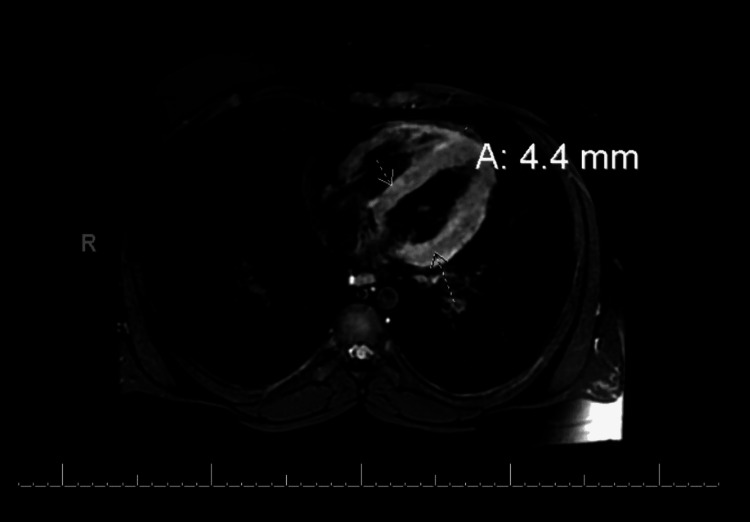
Cardiac MRI The image shows patchy mural delayed myocardial enhancement with relative sparing of the endocardium within the septum, and inferior and lateral walls at the cardiac base MRI: magnetic resonance imaging

Cardiac enzymes were noted to be elevated, with a troponin of 1.39 ng/mL (0.00-0.01), total creatine kinase of 900 U/L (39-308), creatine kinase-MB of 73.3 ng/mL (0.0-3.6) and a creatine kinase-MB index of 8.1 (0.0-2.8). A lipid panel was performed, with no abnormal results noted. The EKG showed normal sinus rhythm with ST-segment elevation in some lateral leads (Figure 1). White blood cell count, C-reactive protein, and D-dimer were elevated at 11.0 K/ul (5.0-10.0), 160 mg/L (0-10), and 0.70 ug/mL (0.00-0.40) respectively. The echocardiogram revealed an ejection fraction (EF) of 60-65% and a right ventricular systolic pressure (RVSP) of 30-35 mmHg. Chest X-ray and CT angiography (CTA) of the chest were negative for any acute changes. He tested negative on the COVID-19 molecular testing while on admission. Cardiac MRI revealed patchy mural delayed myocardial enhancement with relative sparing of the endocardium within the septum, and inferior and lateral walls at the cardiac base (Figure 2). The patient was managed with steroids and Tylenol as needed for pain management. He was also placed on telemetry for rhythm monitoring. Cardiac enzymes were monitored over the course of the hospitalization and were seen to have plateaued. Therefore, with the resolution of symptoms and the decreasing need for analgesics, the patient was discharged home. He was followed up a month later as an outpatient and was found to be stable. The patient will be seen in the clinic in six months and subsequently every year, provided he continues to remain at baseline.

## Discussion

The development of COVID-19 vaccines by various pharmaceutical companies has resulted in a reduction in morbidity and mortality from COVID-19 infection [6]. While these vaccines have been associated with some complications, their benefits have been proven to outweigh the risks [6]. Though rare, myocarditis has been reported following COVID-19 vaccination [2]. The United States Centers for Disease Control and Prevention has reported a prevalence of about 12.6 cases per million doses of second-dose mRNA vaccine in individuals aged 12-39 years, with a male predominance [2]. Although the exact mechanism behind COVID-19 vaccine-induced myocarditis is not well understood, a number of theories have been proposed [2]. Some of these postulations include molecular mimicry between the mRNA vaccine spike protein and self-antigen, immunological response to the mRNA vaccines, triggers from already dysregulated immunologic pathways, and poorly regulated expression of cytokines [2]. The basis behind the male sex predominance may be related to the difference in immune response with sex hormones and myocarditis and the fact that cardiac disease in women is usually under-diagnosed and under-reported [2]. We presented a case report on myocarditis as a rare complication of COVID-19 vaccines.

Our patient was an 18-year-old male with two previous episodes of COVID-19 infection who presented with chest pain, fever, cough, and shortness of breath after receiving the first dose of the Moderna vaccine. He also had other symptoms such as fatigue, nausea, diarrhea, and nose bleeds, which may likely have been the other side effects of the vaccine. However, on presentation, he had an elevated cardiac enzyme with the EKG showing some ST-segment elevations in some of the lateral leads. Cardiac MRI was suggestive of myocarditis. Similar cases have been reported with a majority of them occurring after the second dose of vaccination [6]. However, only a few cases have been observed following the first dose of the Moderna vaccine with or without previous COVID-19 infections [7,8].

A systematic analysis was conducted recently on COVID-19 vaccines and myocarditis. A pooled analysis of the available data showed that COVID-19 vaccine-induced myocarditis was more common in young males, with most of the cases occurring after the second dose and most of them resolving after six days [6]. Findings from the study revealed that 60%, 33%, and 7% of the cases followed the Pfizer-BioNTech vaccine, Moderna vaccine, and Johnson and Johnson vaccine respectively [6]. Also, it was found that while about 67% of the cases occurred after the second dose of the Pfizer-BioNTech vaccine, all the cases of myocarditis associated with the Moderna vaccine occurred after the second dose [6]. However, there was no case of vaccine-induced myocarditis in a patient with a previous COVID-19 infection in that study [6].

Recently, a study on the association between myocarditis and COVID-19 vaccination reported that one out of the four cases have experienced myocarditis 25 days after the first dose of the Moderna vaccine with one prior COVID-19 infection [7]. However, our patient experienced myocarditis eight hours after receiving the vaccine, after two prior COVID-19 infections. Thus, this is the first reported case of vaccine-induced myocarditis following the first dose of the Moderna vaccine in a patient with two previous COVID-19 infections. The susceptibility to developing myocarditis after the first dose of vaccination is likely due to the antibodies developed against the viral antigen from previous infections. The fact that our patient had two different episodes of COVID-19 infection may account for the earlier onset of the myocarditis after the vaccination compared to others. This likely indicates a possible additive effect of antibodies produced from the previous COVID-19 infections. Hence, there is a need for an observational study to better elucidate this assumption.

## Conclusions

COVID-19 vaccines can lead to myocarditis in rare cases. Although most cases occur after the second dose of vaccination, a high index of suspicion is needed when patients with previous COVID-19 infections present with symptoms suggestive of myocarditis, even after the first dose of vaccine. Also, the onset of myocarditis within 24 hours post first dose of vaccination should be anticipated in individuals with two or more previous COVID-19 infections.
